# Risk of severe obesity development: Examining the role of psychological well‐being related measures and sociodemographic factors in two longitudinal UK cohort studies

**DOI:** 10.1111/bjhp.12798

**Published:** 2025-04-21

**Authors:** I Gusti Ngurah Edi Putra, Sam Wilkinson, Michael Daly, Eric Robinson

**Affiliations:** ^1^ Department of Public Health, Policy and Systems, Institute of Population Health University of Liverpool Liverpool UK; ^2^ Department of Psychology, Institute of Population Health University of Liverpool Liverpool UK; ^3^ Department of Psychology Maynooth University Maynooth Ireland

**Keywords:** longitudinal study, mental health, psychological well‐being, severe obesity, weight change

## Abstract

**Objective:**

To examine the prospective association between psychological well‐being related measures and severe obesity development in young and middle‐aged UK adults.

**Design:**

A longitudinal analysis of two cohort studies.

**Methods:**

We used data from the 1958 National Child Development Study (NCDS) and the 1970 British Cohort Study (BCS) to examine the association between baseline psychological well‐being related measures (depressive symptoms, life satisfaction and self‐efficacy) and severe obesity development (defined as body mass index – BMI ≥35 kg/m^2^) and residualized BMI change scores at follow‐up. We analysed repeated measures of baseline and follow‐up pairs with 6‐ to 7‐year follow‐up on average (*n* = 22,390 and 23,811 observations in NCDS and BCS, respectively) using panel data logistic and linear models controlling for sociodemographic factors. We conducted additional analyses using analytical sample sizes with longer follow‐up (16–17 years).

**Results:**

Although a range of sociodemographic factors (e.g., being female, non‐married) were associated with increased risk of severe obesity development, we found limited evidence that psychological well‐being related measures were associated with severe obesity development across cohorts and pooled analyses. Depressive symptoms, life satisfaction and self‐efficacy were, however, associated with relatively small changes in continuous BMI change across analyses, and this tended to be limited to participants without obesity (BMI 18.5 to <30 kg/m^2^) and not those already living with obesity (BMI 30 to <35 kg/m^2^) at baseline.

**Conclusions:**

There is limited evidence that psychological well‐being related measures prospectively predict the development of severe obesity. Poorer psychological well‐being is associated with modest changes in body weight in individuals without obesity.

## INTRODUCTION

Obesity is a major public health problem with its global prevalence having doubled since 1990 (WHO, 2024). In England, obesity rates have similarly increased from 15% to 29% over the last three decades (1993–2022) (Stiebahl, 2025). Obesity and its subsequent adverse physical health outcomes are estimated to cost the National Health Service (NHS) £6.5 billion annually in the UK (Department of Health and Social Care, 2024). In particular, living with a higher class of or ‘severe’ obesity (defined as body mass index – BMI ≥35 kg/m^2^, e.g., as in Koliaki et al., 2023) is considered a ‘high risk’ for developing a range of poorer health outcomes, increased risk of mortality and disability (Hoogendoorn et al., 2023; Kyrou et al., 2011; Putra et al., 2024). A study found that although there is a positive association between higher body mass index (BMI) and risk of cardiovascular mortality, only severe obesity was statistically significantly associated with increased risk of CVD mortality when compared to normal body weight (Kee et al., 2017). In addition, an estimation of obesity‐related disease burden between cohorts of people with BMI means of 30 and 35 kg/m^2^ in five European countries, including the UK, showed that the cohort with BMI of 35 kg/m^2^ had lower life expectancy, higher incidence of diabetes and cardiovascular diseases and greater total lifetime health care costs (Hoogendoorn et al., 2023). Furthermore, studies during the COVID‐19 pandemic reported that severe obesity was linked to an escalated risk of poor COVID‐19 outcomes (Hoong et al., 2021) and was an independent risk factor of COVID‐19 mortality in hospitalized young adults (Klang et al., 2020).

The prevalence of severe obesity (BMI ≥35 kg/m^2^) predicted to increase from 10% in 2020 to 20% in 2035 (Koliaki et al., 2023). Because severe obesity is rarely reversed once developed (Fildes et al., 2015), this will substantially contribute to a higher prevalence of obesity‐related diseases and greater loss in quality‐adjusted life years (QALYs), posing greater threats to public health and healthcare systems (Hoogendoorn et al., 2023). Therefore, understanding factors associated with increased risk of developing severe obesity is important to inform current obesity‐related public health policies and interventions. A previous study using electronic health records in England suggested that some sociodemographic factors, such as younger age groups and neighbourhood deprivation, were consistently associated with higher odds of transitioning into a higher BMI group, including the development of very severe obesity (BMI ≥40 kg/m^2^) (Katsoulis et al., 2021). However, there is a dearth of direct evidence on whether psychological well‐being related measures are associated with severe obesity development, which may have implications for how obesity is clinically managed and treated.

Findings from reviews and meta‐analyses demonstrate bidirectional associations between obesity and poor psychological well‐being (Blaine, 2008; Luppino et al., 2010; Mannan et al., 2016; Steptoe & Frank, 2023). Living with obesity is associated with an increased risk of depression (Luppino et al., 2010), partly explained by exposure to chronic stress, such as weight stigma (Robinson et al., 2017; Tomiyama, 2014) and systemic low‐grade inflammation, gauged by C‐reactive protein (CRP) levels (Chu et al., 2023). Conversely, depression is also prospectively linked to obesity (Blaine, 2008; Luppino et al., 2010; Mannan et al., 2016; Shell et al., 2024). Poor psychological well‐being may contribute to weight gain and/or obesity development through emotional eating and physical inactivity (Ibáñez Román et al., 2023; Lazarevich et al., 2016; Matta et al., 2019; Paans et al., 2018; Zager Kocjan et al., 2024). In addition, biological responses to poor psychological well‐being and chronic stress, such as increased hypothalamic–pituitary‐adrenocortical (HPA) axis activation and associated cortisol secretion, have been suggested to increase appetite and fat accumulation (Anagnostis et al., 2009; Kappes et al., 2023; Lucassen & Cizza, 2012; Schvey et al., 2014; Tomiyama, 2014).

While poor psychological well‐being is prospectively linked to obesity development (Blaine, 2008; Luppino et al., 2010; Mannan et al., 2016; Shell et al., 2024), limited evidence exists on the extent to which poorer psychological well‐being is also responsible for severe obesity development. Contemporary psychosocial models of obesity indicate that exposure to chronic stress (e.g., weight stigma) and poor psychological well‐being may explain why obesity harms physical health and may be hard to reverse (Tomiyama, 2014, 2019). A study of US adults found that exposure to weight stigma is prospectively associated with increased odds of remaining in the obesity weight category over a 4‐year follow‐up (Sutin & Terracciano, 2013). Further weight gain linked to poor psychological well‐being may occur as emotional eating, a common coping mechanism for poor psychological well‐being, is prevalent among individuals with overweight and obesity (Chew et al., 2025). In addition, worse psychological well‐being is more common among individuals with severe obesity (Putra et al., 2024; Scott et al., 2008), which may suggest a key role for psychological well‐being in explaining severe obesity development, but this has not been formally tested. Yet it has also been argued that poorer psychological well‐being may not have an additional contribution to weight gain once obesity is already developed, as one study to date failed to find an association between poor psychological well‐being and increased BMI in individuals living with obesity (Cloostermans et al., 2015). Building on these contrasting propositions, we aimed to examine whether measures of psychological well‐being are associated with severe obesity development across adulthood.

In the present study, we investigated the role of psychological well‐being related measures in explaining the onset of severe obesity in young and middle‐aged adults. We used two comparable British birth cohort studies, the 1958 National Child Development Study (NCDS) and the 1970 British Cohort Study (BCS). Both are national cohort studies that have collected data on psychological well‐being related measures and BMI across multiple waves from young to middle‐aged adults (≤50 years), enabling us to examine the role of poor psychological well‐being in the development of severe obesity. We excluded data collected in older adulthood (>50 years) as cohort members may experience unintentional weight loss due to undiagnosed health conditions, which may further be associated with poorer psychological well‐being (McMinn et al., 2011), resulting in concerns over reverse causality introduced by unmeasurable confounding (chronic illness) (Tobias, 2017). The exclusion of older adults is relevant to the notion of ‘obesity paradox’ where obesity has been reported as a protective factor against depressive symptoms in older adults (Yu et al., 2022), potentially due to reverse causation bias.

We examined associations between psychological well‐being and the development of severe obesity over 4–17 years of follow‐up in the NCDS and BCS birth cohort studies. Psychological well‐being was measured by depressive symptoms because of its previously studied association with obesity (BMI ≥30 kg/m^2^) onset (Blaine, 2008; Luppino et al., 2010; Mannan et al., 2016; Shell et al., 2024) and other available measures previously tested as psychological predictors of weight‐related outcomes: life satisfaction (Korkeila et al., 1998) and self‐efficacy (Nezami et al., 2016). Previous studies used either NCDS or BCS or both to understand the association between obesity and psychological well‐being (Geoffroy et al., 2014; Mulugeta et al., 2018; Scarpato et al., 2021; White et al., 2012), but no studies to date have investigated the extent to which psychological factors are prospectively associated with severe obesity development.

## METHODS

We pre‐registered our analysis approach at https://doi.org/10.17605/OSF.IO/2QEPT.

### Study design and data

Data came from two UK cohort studies with participants born 12 years apart, NCDS and BCS (see below). Both cohort studies received ethics approval from the Multicentre Research Ethics Committee, and informed consent was obtained from all participants.

#### The 1958 National Child Development Study (NCDS)

NCDS was started as the Perinatal Mortality Survey, recruiting more than 17,000 people born in England, Wales and Scotland in a single week of 1958 (Power & Elliott, 2005). The first follow‐up for cohort members was conducted at age 7 (1965) and their information on socioeconomic status, health, social circumstances, and well‐being has been collected until age 62 (2020–2024). For this study, we used data from cohort members at ages 23, 33, 42 and 50, representing a period from young to middle adulthood. Measures of weight status and psychological well‐being were available for all studied waves.

#### The 1970 British Cohort Study (BCS)

BCS recruited around 17,000 people born in England, Wales and Scotland in a single week in 1970 (Elliott & Shepherd, 2006). Cohort members were followed up from ages 5 (1975) to 51 (2021–2024), and their information on socioeconomic circumstances, health and well‐being, among other factors, has been documented. For this study, we selected waves where cohort members' lives span from young to middle adulthood (ages 26, 30, 34, 42 and 46) and data on psychological well‐being related measures and weight status were available.

### Variables

#### Weight status

In both NCDS and BCS, BMI in kg/m^2^ was mostly determined based on self‐reported measures of height and weight (see Johnson et al., 2015). For both cohorts, we used harmonized BMI data (CLOSER, 2022) and the details are available elsewhere (Johnson et al., 2015). As the present study investigated the development of severe obesity (BMI ≥35 kg/m^2^), we only included participants with non‐severe obesity (BMI 18.5 to < 35 kg/m^2^) at baseline. Participants with underweight (BMI < 18.5 kg/m^2^) at baseline and follow‐up were excluded, as being underweight is associated with poorer psychological well‐being (Geoffroy et al., 2014).

Out of a total of 23,734 eligible observations (from ages 23, 33, 42 and 50) that had baseline and follow‐up weight status in NCDS, we excluded 1344 observations because of having underweight at baseline or follow‐up (*n* = 778) and severe obesity at baseline (*n* = 566), making a final maximum analytical sample size of 22,390 observations. For BCS, from a total of 25,308 eligible observations (ages 26, 30, 34, 42 and 46), 1497 observations were excluded due to having underweight at baseline or follow‐up (*n* = 613) and severe obesity at baseline (*n* = 884), leaving a maximum analytical sample size of 23,811 observations.

#### Psychological well‐being related measures

We included three psychological well‐being‐related measures available in NCDS and BCS (see Dodgeon, 2018; McElroy et al., 2020). We follow a previous study (Putra et al., 2025) to quantify psychological well‐being‐related measures and convert them into *z*‐scores to allow comparisons across measures.

##### Depressive symptoms

Following a previous study using both cohorts (Arias‐de la Torre et al., 2021), depressive symptoms were defined using a shorter nine‐item version of the Malaise Inventory (McGee et al., 1986; Rodgers et al., 1999). This scale has been used to assess levels of depressive symptoms and psychological distress in the general population (Gondek et al., 2022; Rodgers et al., 1999). Cohort members were asked to respond to nine items (e.g., ‘Do you feel tired most of the time?’, ‘Do you often feel miserable or depressed?’) with dichotomous answers (yes, no). A total score ranging from 0 to 9 was generated by adding together all the responses with a higher score indicating greater depressive symptoms. We found that this 9‐item measure of depressive symptoms had good internal consistency (Cronbach's alpha = .75). As depressive symptoms were collected from young adulthood, the analysis for this psychological measure included all waves from ages 23 in NCDS and 26 in BCS. Therefore, sample sizes for examining depressive symptoms were the same as the maximum analytical sample size for each cohort (22,390 and 23,811 observations for NCDS and BCS, respectively).

##### Life satisfaction

Life satisfaction in both cohorts was measured through cohort members' responses to this single question, ‘How dissatisfied or satisfied you are about the way your life has turned out so far?’ with responses from 0 = ‘completely dissatisfied’ to 10 = ‘completely satisfied’ (e.g., as in Flèche et al., 2021; Hatch et al., 2010). A higher score indicates greater life satisfaction. Life satisfaction was not collected at age 23 in NCDS (see Flèche et al., 2021), and therefore, the analysis for this psychological measure included waves from ages 33 in NCDS and 26 in BCS. The maximum analytical sample size for analysing life satisfaction was 14,453 for NCDS and 23,811 for BCS.

##### Self‐efficacy

Following a previous study (Hatch et al., 2010), we quantified self‐efficacy across cohorts using three dichotomous items: ‘I usually have a free choice and control over my life’, ‘I usually get what I want out of life’, and ‘Usually I can run my life more or less as I want to’. Responses were totalled to create a summary score ranging from 0 to 3 with a higher score indicating greater self‐efficacy. This three‐item measure was observed to have acceptable internal consistency (Cronbach's alpha = .63). Self‐efficacy items were collected from ages 33 in NCDS and 30 in BCS, and therefore, the analysis was restricted from these waves, making the maximum analytical sample size of 14,453 for NCDS and 18,565 for BCS.

#### Covariates

Age (based on waves of data collection: 23, 33, 42, 50 in NCDS; and 26, 30, 34, 42 and 46 in BCS), sex (female, male), ethnicity (White and non‐White (e.g., as in Geoffroy et al., 2014)), and marital status (single, married and others (e.g., as in Arias‐de la Torre et al., 2021)) were adjusted in the analysis. We also controlled for socioeconomic status (SES), including childhood SES, time‐varying cohort members' occupation, education and housing tenure (e.g., as in Putra et al., 2025). Childhood SES representing social class was defined based on the father's occupation, classified into five categories: occupational group (OG) 1 for professional occupations, OG 2 for managerial and technical occupations, OG 3 for skilled occupations, OG 4 for partly skilled occupations, and OG 5 for unskilled occupations (e.g., as in Daly & Egan, 2017). Cohort members' current or last occupation was also determined following the above occupational categories. Educational attainment reported by cohort members was grouped following National Vocational Qualification (NVQ) rankings from NVQ‐1 for second‐level or vocational education to NVQ‐5 for postgraduate degrees (e.g., as in Daly & Egan, 2017). In addition to social class (occupation) and education, we also controlled for housing tenure, categorizing cohort members as owner‐occupier or other (e.g., as in Geoffroy et al., 2014), as this potentially acts as a proxy measure of accumulated wealth (Connolly et al., 2010). Housing tenure has been reported to be strongly associated with weight status (Tranter & Donoghue, 2017), psychological well‐being (Angel & Gregory, 2023; Ellaway et al., 2016) and CRP levels (a biomarker indicating inflammation due to stress and infection) (Clair & Hughes, 2019), independently of occupation and/or education.

### Data analysis

#### Main analysis

We investigated the role of psychological well‐being related measures separately in explaining severe obesity development (vs. no severe obesity development). We performed a binary logistic regression panel data analysis implemented using the ‘xtlogit’ command in STATA with an option of ‘vce (cluster participant_ID)’ added to obtain robust standard errors by clustering observations within each participant's unique ID. We used this approach to conduct a pooled analysis of baseline and follow‐up pairs within each cohort (e.g., as in Geoffroy et al., 2014; Putra et al., 2025). We examined the longitudinal associations between time‐varying psychological well‐being related measures at baseline in predicting subsequent severe obesity development (vs. no development) from baseline to follow‐up, controlling for time‐varying and non‐varying covariates and baseline BMI.

In NCDS, time‐varying psychological measures at ages 23, 33 and 42 predicted subsequent weight change outcomes at ages 33, 42 and 50, respectively. In BCS, time‐varying psychological measures at ages 26, 30, 34 and 42 predicted subsequent weight change outcomes at ages 30, 34, 42 and 46, respectively. As some psychological well‐being‐related measures were not collected in the earliest wave (see sub‐section ‘Psychological well‐being related measures’), we analysed different numbers of waves in each cohort to accommodate various psychological well‐being‐related measures. Associations between psychological well‐being‐related measures and severe obesity development were estimated by odds ratio (OR) along with 95% confidence intervals (CI) and *p*‐value.

To avoid further sample loss due to listwise deletion, missing information on psychological well‐being related measures on relevant baselines and sociodemographic covariates was estimated using multiple imputations by chained equations (MICE) (Azur et al., 2011; White et al., 2011). Based on the maximum analytical sample sizes by cohorts and psychological well‐being related measures (see sub‐section ‘Psychological well‐being related measures’), the proportion of missing observations ranged from 11% to 23%. We used STATA (‘mi impute chained’) (Berglund, 2014; Manly & Wells, 2015; Woods et al., 2021) to create 20 imputed datasets. All variables included in the analysis were fitted in the imputation model (e.g., as in Arias‐de la Torre et al., 2021). We also included auxiliary variables (birth weight of cohort members, mother's age at the time of birth, smoking status during pregnancy and breastfeeding habits (e.g., as in Khanolkar & Patalay, 2021; Putra et al., 2025)) to improve the imputation model. Using MICE, the final sample size was the same as the maximum analytical sample size by cohorts and psychological well‐being related measures (see sub‐section ‘Psychological well‐being related measures’). The significance level for the main analysis above was set at *p* < .05. To account for multiple tests, we used the Benjamini–Hochberg (BH) adjustment approach (Benjamini & Hochberg, 1995) for the following additional/sensitivity analysis.

#### Additional/sensitivity analysis

We examined whether the findings were consistent when the outcomes were fitted as continuous variables, presented as residualized BMI change scores between baseline and follow‐up (as opposed to the binary outcome), determined from regressing follow‐up BMI by baseline BMI (e.g., as in Deforche et al., 2015; Putra et al., 2025). This analysis was conducted using the same analytical sample size of severe obesity development in each cohort. We used linear regression analysis implemented using ‘xtreg’ command in STATA to fit a continuous outcome.

Furthermore, we replicated the main analysis above to test a longer period of follow‐up (e.g., as in Putra et al., 2025). We selected the earliest cohort wave in which all psychological measures of interest were available (ages 33 in NCDS, 30 in BCS) and predicted the outcomes (severe obesity development, residualized BMI change scores) of the last available follow‐up (ages 50 in NCDS, 46 in BCS), in order to provide a comparable long‐term follow‐up (16–17 years across adulthood) for the same psychological measures across cohorts. Therefore, psychological well‐being related measures had the same analytical sample size, and this would also allow us to examine evidence of multiple psychological measures predicting the outcomes if we found some measures were statistically significant in the individual models. We used logistic and linear regression models.

For each outcome (severe obesity development, residualized BMI change scores), we conducted a cohort‐pooled analysis to create an overall estimate for each psychological well‐being related measure. We also added a two‐way interaction term between psychological well‐being related measures and cohort (BCS vs. NCDS) in the cohort‐pooled analysis to examine whether the association between psychological well‐being related measures and the outcomes was stronger for a particular cohort (e.g., as in Putra et al., 2025).

## RESULTS

Based on Table 1, while NCDS included similar proportions of males (51%) and females (49%), BCS included slightly more females (54%) than males (46%). Across cohorts: the majority were White (99%), married (50%–63%), had normal body weight (57%–65%), worked in intermediate or skilled occupations (67%) (as did their fathers) and owned their homes (63%–68%). The proportion of severe obesity development at follow‐up was 3% in NCDS and BCS.

**TABLE 1:** Baseline characteristics of participants from the 1958 NCDS and 1970 BCS birth cohorts.

Variables	NCDS		BCS	
	*n* = 22,390^a^	%	*n* = 23,811^a^	%
Sex				
Male	11,332	50.61	10,967	46.06
Female	11,058	49.39	12,844	53.94
Age (years)				
23	7937	35.45	*N/A*	*N/A*
26	*N/A*	*N/A*	5246	22.03
30	*N/A*	*N/A*	7369	30.95
33	7886	35.22	*N/A*	*N/A*
34	*N/A*	*N/A*	5888	24.73
42	6567	29.33	5308	22.29
Ethnicity				
White	22,271	99.47	23,613	99.17
Non‐White	64	.29	124	.52
*Missing*	*55*	.*25*	*74*	.*31*
Marital status				
Single	6011	26.85	9887	41.52
Married	14,087	62.92	11,951	50.19
Separated/divorced/widowed	2073	9.26	1885	7.92
*Missing*	*219*	.*98*	*88*	.*37*
Childhood SES (father's occupation)				
Professional	962	4.30	1210	5.08
Intermediate	4070	18.18	5546	23.29
Skilled	9969	44.52	10,528	44.21
Partly skilled	2683	11.98	2477	10.40
Unskilled	1505	6.72	1095	4.60
Others	777	3.47	566	2.38
*Missing*	*2424*	*10.83*	*2389*	*10.03*
Cohort member's occupation				
Professional	928	4.14	1326	5.57
Intermediate	5590	24.97	7842	32.93
Skilled	9469	42.29	8155	34.25
Partly skilled	2761	12.33	2223	9.34
Unskilled	662	2.96	384	1.61
Others	2980	13.31	3881	16.30
Cohort member's education				
No qualification	2246	10.03	2290	9.62
NVQ level 1	2605	11.63	1858	7.80
NVQ level 2	7401	33.05	7004	29.41
NVQ level 3	3404	15.20	3531	14.83
NVQ level 4	2837	12.67	7665	32.19
NVQ level 5	2544	11.36	1457	6.12
*Missing*	*1353*	*6.04*	*6*	*.03*
Housing tenure				
Owner‐occupier	14,026	62.64	16,297	68.44
Other	7550	33.72	7386	31.02
*Missing*	*814*	*3.64*	*128*	*.54*
Baseline BMI (in kg/m^2^) mean (SD)				
Pooled BMI across baselines		24.21 (3.35)		24.85 (3.58)
23	7937	22.81 (2.76)	*N/A*	*N/A*
26	*N/A*	*N/A*	5246	23.62 (3.20)
30	*N/A*	*N/A*	7369	24.63 (3.49)
33	7886	24.72 (3.32)	*N/A*	*N/A*
34	*N/A*	*N/A*	5888	25.27 (3.57)
42	6567	25.32 (3.47)	5308	25.92 (3.66)
Baseline BMI category				
Normal weight	14,443	64.51	13,582	57.04
Overweight	6413	28.64	7796	32.74
Non‐severe obesity	1534	6.85	2433	10.22
Severe obesity development				
Yes	723	3.23	817	3.43
No	21,667	96.77	22,994	96.57

*Note:* The italic indicates N/A and missing values.

Abbreviations: %, percentage; N/A, not available as data were not collected at those ages (see below); NVQ, National Vocational Qualification; SD, standard deviation.

^a^ Number of eligible observations, excluding participants with underweight (BMI < 18.5 kg/m^2^) at baseline and follow‐up, and severe obesity (BMI ≥ 35 kg/m^2^) at baseline (NCDS combined baseline data from ages 23, 33 and 42; BCS combined baseline data from ages 26, 30, 34 and 42).

We present associations between psychological well‐being related measures and weight outcomes controlling for sociodemographic characteristics. Findings from our main analysis (Table 2) indicate no evidence for psychological well‐being related measures in predicting the development of severe obesity (vs. no development) in NCDS and BCS. However, we found statistically significant associations between all the psychological well‐being related measures and BMI change in BCS participants and non‐significant but directionally similar results in NCDS (Table 3). While higher depressive symptoms were significantly associated with increased BMI (*β* = .05, 95% CI = .02, .08), greater life satisfaction (*β* = −.07, 95% CI = −.10, −.04) and self‐efficacy (*β* = −.06, 95% CI = −.10, −.02) were linked to significantly decreased BMI in BCS.

**TABLE 2:** Associations between individual psychological well‐being‐related measures and severe obesity development (vs. no development), controlling for sociodemographic covariates.

Psychological well‐being related measures	Severe obesity development vs. no development							
	NCDS				BCS			
	*n*	OR	95% CI	*p*‐Value	*n*	OR	95% CI	*p*‐Value
Depressive symptoms	22,390	1.00	.92, 1.08	.968	23,811	.99	.91, 1.08	.882
Life satisfaction	14,453	.97	.86, 1.09	.577	23,811	.97	.89, 1.05	.401
Self‐efficacy	14,453	.99	.89, 1.12	.918	18,565	.99	.91, 1.08	.862

*Note*: Only participants with no severe obesity (BMI 18.5 to <35 kg/m^2^) at baseline were included. All psychological well‐being related measures were in *z*‐scores (mean = 0; SD = 1). Separate regression models were developed for each psychological well‐being related measure, adjusted for age, sex, ethnicity, marital status, childhood SES (or father's occupation), participant's occupation, education, housing tenure and baseline BMI.

Abbreviations: CI, confidence intervals; *n*, number of observations; OR, odds ratio.

**TABLE 3:** Associations between individual psychological well‐being related measures and residualized BMI change scores based on the analytical sample size of severe obesity development, controlling for sociodemographic covariates.

Psychological well‐being related measures	Changes in BMI							
	NCDS				BCS			
	*n*	*β*	95% CI	*p*‐Value	*n*	*β*	95% CI	*p*‐Value
Depressive symptoms	22,390	.03	−.01, .07	.117	23,811	.05	.02, .08	.001*
Life satisfaction	14,453	−.05	−.09, .00	.050	23,811	−.07	−.10, −.04	<.001*
Self‐efficacy	14,453	−.01	−.06, .03	.571	18,565	−.06	−.10, −.02	.003*

*Note*: Only participants with no severe obesity (BMI 18.5 to <35 kg/m^2^) at baseline were included. All psychological well‐being related measures were in *z*‐scores (mean = 0; SD = 1). Separate regression models were developed for each psychological well‐being related measure, adjusted for age, sex, ethnicity, marital status, childhood SES (or father's occupation), participant's occupation, education and housing tenure.

Abbreviations: CI, confidence intervals; *n*, number of observations; *β*, regression coefficient.

* *p*‐Value remained statistically significant after correcting multiple comparisons (see Table S8).

We conducted additional analyses over a longer period of follow‐up (16 or 17 years) by fitting regression models using the first wave where all the psychological well‐being related measures were collected as baseline and the last wave as follow‐up (ages 33 and 30 as baseline and ages 50 and 46 as follow‐up in NCDS and BCS, respectively). An increase in life satisfaction scores by one SD was associated with lower odds of severe obesity development in BCS participants (OR = .84, 95% CI = .74, .96), but not NCDS (Table 4). Other psychological well‐being measures were not associated with severe obesity development in NCDS or BCS (Table 4). When the continuous BMI outcome was examined, depressive symptoms were associated with increased BMI in both NCDS (*β* = .13, 95% CI = .05, .21) and BCS (*β* = .18, 95% CI = .08, .28) and life satisfaction was associated with decreased BMI in BCS (*β* = −.17, 95% CI = −.27, −.07) (Table 5).

**TABLE 4:** Associations between individual psychological well‐being related measures and severe obesity development (vs. no development) for a longer follow‐up, controlling for sociodemographic covariates.

Psychological well‐being related measures	Severe obesity development vs. no development							
	NCDS baseline: Age 33, follow‐up: Age 50				BCS baseline: Age 30, follow‐up: Age 46			
	*n*	OR	95% CI	*p*‐value	*n*	OR	95% CI	*p*‐value
Depressive symptoms	6018	1.13	1.00, 1.29	.060	5517	1.06	.95, 1.18	.294
Life satisfaction	6018	.84	.74, .96	.012*	5517	.94	.85, 1.05	.283
Self‐efficacy	6018	.89	.79, 1.01	.070	5517	1.03	.93, 1.15	.590

*Note*: Only participants with no severe obesity (BMI 18.5 to <35 kg/m^2^) at baseline were included. All psychological well‐being related measures were in *z*‐scores (mean = 0; SD = 1). Separate regression models were developed for each psychological well‐being related measure, adjusted for age, sex, marital status, childhood SES (or father's occupation), participant's occupation, education, housing tenure and baseline BMI. Ethnicity was not controlled for due to a small percentage of participants from ethnic minorities.

Abbreviations: CI, confidence intervals; *n*, number of observations; OR, odds ratio.

* *p*‐Value remained statistically significant after correcting multiple comparisons (see Table S8).

**TABLE 5:** Associations between individual psychological well‐being related measures and residualized BMI change scores based on the analytical sample size of severe obesity development for a longer follow‐up, controlling for sociodemographic covariates.

Psychological well‐being related measures	Changes in BMI							
	NCDS baseline: Age 33, follow‐up: Age 50				BCS baseline: Age 30, follow‐up: Age 46			
	*n*	*β*	95% CI	*p*‐Value	*n*	*β*	95% CI	*p*‐Value
Depressive symptoms	6018	.13	.05, .21	.002*	5517	.18	.08, .28	<.001*
Life satisfaction	6018	−.07	−.15, .02	.120	5517	−.17	−.27, −.07	.001*
Self‐efficacy	6018	−.10	−.18, −.02	.019	5517	−.09	−.19, .01	.080

*Note*: Only participants with no severe obesity (BMI 18.5 to <35 kg/m^2^) at baseline were included. All psychological well‐being related measures were in *z*‐scores (mean = 0; SD = 1). Separate regression models were developed for each psychological well‐being related measure, adjusted for age, sex, marital status, childhood SES (or father's occupation), participant's occupation, education and housing tenure. Ethnicity was not controlled for due to a small percentage of participants from ethnic minorities.

Abbreviations: CI, confidence intervals; *n*, number of observations; *β*, regression coefficient.

* *p*‐Value remained statistically significant after correcting multiple comparisons (see Table S8).

When full‐analytical sample sizes from both cohorts were combined, psychological well‐being related measures remained not associated with severe obesity development, but were associated with BMI change (Table S1). Higher depressive symptoms (*β* = .04, 95% CI = .02, .06) were associated with increased BMI, whilst greater life satisfaction (*β* = −.06, 95% CI = −.08, −.03) and self‐efficacy (*β* = −.04, 95% CI = −.07, −.01) were associated with decreased BMI. Findings from interaction analysis between psychological well‐being related measures and study cohorts (BCS vs. NCDS) in predicting the outcomes (severe obesity development, residualized BMI change scores) indicated that the effect sizes of the associations were not statistically different between cohorts for both outcomes of interest (Table S2).

Relevant pooled analysis was also conducted for analytical sample sizes with longer follow‐up (Table S3). We found that life satisfaction was associated with reduced odds of severe obesity development (OR = .90, 95% CI = .83, .98) and all the psychological well‐being related measures: depressive symptoms (*β* = .15, 95% CI = .08, .21), life satisfaction (*β* = −.12, 95% CI = −.18, −.06), self‐efficacy (*β* = −.09, 95% CI = −.15, −.03), were associated with BMI change at follow‐up. Findings from interaction analysis showed only a statistically significantly larger effect size for depressive symptoms in predicting an increase in BMI for BCS compared to NCDS (Table S4). As we found all the psychological well‐being related measures were associated with BMI change in individual models (Table S3), we conducted additional analysis by including all these measures in the same model (Table S5). We found that only depressive symptoms were associated with BMI change independently of other psychological well‐being related measures.

While psychological well‐being related measures did not largely play an important role in the development of severe obesity (as opposed to continuous BMI change outcome), we observed some sociodemographic characteristics associated with the severe obesity development risk in the pooled analysis of full‐analytical sample sizes (Table S6). Older ages (vs. age 23) at baseline were associated with lower odds of severe obesity development. Being female (vs. male) and not currently married (either single or others) (vs. married) were at increased risk of developing severe obesity. We did not find evidence that poorer SES was associated with risk of developing severe obesity.

### Exploratory analyses

Having confirmed stronger associations between poorer psychological well‐being and increased BMI than with severe obesity development (vs. no development) across analyses, we hypothesized that this may be because poorer psychological well‐being is related to the risk of weight gain prior to obesity development, but not further escalation of obesity. To examine this hypothesis, we conducted non‐pre‐registered analysis to examine the contribution of poorer psychological well‐being to weight gain in people with and without obesity at baseline. We used the pooled full‐analytical sample size from both cohorts (see Table S1) due to having greater analytical power to conduct stratified analysis by baseline BMI categories: non‐obesity (BMI 18.5 to <30 kg/m^2^) and non‐severe obesity (BMI 30 to <35 kg/m^2^) (Table S7). In participants without obesity at baseline (BMI 18.5 to <30 kg/m^2^), poorer psychological well‐being related measures were consistently associated with increased BMI, similar to the findings reported in Table S1. However, our analysis of participants with non‐severe obesity (BMI 30 to <35 kg/m^2^) at baseline showed no psychological well‐being related measures associated with BMI change. These findings suggest that although psychological well‐being related measures explain weight gain prior to obesity, they do not relate to weight gain among those living with obesity.

## DISCUSSION

We found limited evidence of a prospective link between psychological well‐being related measures and severe obesity development in separate and pooled analyses of full‐analytical sample sizes. However, we did find that life satisfaction was linked to a reduced likelihood of developing severe obesity in an analytical sample size with a longer follow‐up of NCDS, but not BCS. Poor psychological well‐being related measures were consistently associated with modest increases in BMI (continuous measure) across analyses. In particular, depressive symptoms were linked to increased BMI, independently of other psychological well‐being related measures. The association between poorer psychological well‐being and increased BMI was only evident in participants without obesity (BMI 18.5 to <30 kg/m^2^), but not in those with non‐severe obesity (BMI 30 to <35 kg/m^2^) at baseline. Collectively, these findings suggest that although the studied psychological well‐being factors may explain the risk of weight gain prior to the development of obesity, they are not important in explaining the subsequent development of severe obesity. Instead, sociodemographic factors such as gender and marital status may be more important in predicting the risk of severe obesity development.

Psychological well‐being (e.g., depressive symptoms) and chronic stress (e.g., weight stigma) have been noted as a contributor to weight gain (Blaine, 2008; Luppino et al., 2010; Robinson et al., 2020; Tomiyama, 2014). To extend the current literature, we examined whether poorer psychological well‐being is linked to the development of severe obesity. We found limited evidence for depressive symptoms, life satisfaction and self‐efficacy consistently being associated with severe obesity development. However, lower life satisfaction was associated with higher odds of severe obesity development over a 16/17‐year follow‐up analysis in NCDS (but not BCS) and in the pooled analysis of both cohorts. The significant association for life satisfaction only appeared for longer follow‐up (16/17 years), but not for shorter follow‐up (6–7 years on average). A previous study also demonstrated a statistically significant association between low life satisfaction and increased risk of weight gain over a longer follow‐up period (15 years), but not over a shorter follow‐up period (6 years) in an additional sub‐sample analysis specific to young adult women (Korkeila et al., 1998). This may indicate a potential ‘delayed effect’ where life satisfaction may be linked to reduced risk of severe obesity. Specifically, greater life satisfaction is linked to a range of positive behaviours (e.g., physical activity, healthy eating) (Grant et al., 2009; Kim et al., 2021), as well as physiological health indicators (e.g., lower inflammation (Ironson et al., 2018), cortisol levels (Smyth et al., 2017)) which if maintained may contribute to weight gain over several years and then eventually lead to severe obesity development. Nevertheless, the inconsistencies in the present study's findings (longer vs. shorter follow‐up analytical sample sizes) require further investigation.

Although we found depressive symptoms may be the strongest predictor for increased BMI (independently of life satisfaction and self‐efficacy), there was no evidence that depressive symptoms were associated with the development of severe obesity. This may be due to a small average increase in BMI associated with a one‐SD increase score in depressive symptoms observed in the present study (*β* = .04 and .15 kg/m^2^ in the pooled analyses of full (Table S1) and partial analytical (Table S3) sample sizes, respectively). Given that participants had an average baseline BMI of 24 to 25 kg/m^2^ (see Table 1), an average BMI increase of .04 to .15 kg/m^2^ is unlikely to result in many participants developing severe obesity over the follow‐up period.

An earlier study of Dutch participants (Cloostermans et al., 2015) found similar results in which psychological distress was associated with an increase in overall BMI, but did not predict the onset of overweight. The study also observed no BMI change associated with greater psychological distress in participants with obesity at baseline (Cloostermans et al., 2015). In line with this, our exploratory analyses indicated that depressive symptoms and other psychological well‐being related measures predicted BMI change in people without obesity (BMI 18.5 to <30 kg/m^2^), but not in those with non‐severe obesity (BMI 30 to <35 kg/m^2^) at baseline. This may indicate that after obesity onset, poorer psychological well‐being may not be a key factor explaining further weight gain and the development of severe obesity. For example, it may be the case that poorer well‐being earlier in life (prior to severe obesity onset) results in patterns of weight gain–promoting behaviours (e.g., emotional eating, physical inactivity) (Chew et al., 2025; Ibáñez Román et al., 2023; Lazarevich et al., 2016; Matta et al., 2019; Paans et al., 2018; Zager Kocjan et al., 2024), which then become habitual and continue to shape weight trajectories independent of well‐being once severe obesity has developed.

Our findings on associations between poor psychological well‐being and increased BMI were consistent across follow‐up periods. This is in line with previous findings on psychosocial factors associated with weight‐related outcomes, including depressive symptoms (Blaine, 2008; Luppino et al., 2010; Mannan et al., 2016; Shell et al., 2024), life satisfaction (Korkeila et al., 1998) and self‐efficacy (Nezami et al., 2016). Subsequent increased BMI associated with poorer psychological well‐being may be explained by weight gain‐related behavioural mechanisms (e.g., emotional eating, physical inactivity) (Lazarevich et al., 2016; Matta et al., 2019; Paans et al., 2018) and biological mechanisms that increase appetite and fat accumulation (e.g., increased HPA axis and cortisol secretion) (Anagnostis et al., 2009; Kappes et al., 2023; Lucassen & Cizza, 2012; Schvey et al., 2014; Tomiyama, 2014).

Our study also found that other factors may be more important in severe obesity development than our studied psychological well‐being related measures. Specifically, older ages (vs. age 23) at baseline were associated with lower odds of developing severe obesity, while being female (vs. males) and not currently married (vs. married) were linked to a higher likelihood of severe obesity development. Our findings are supported by a previous study in England suggesting that younger ages were associated with increased odds of developing severe obesity (Katsoulis et al., 2021). The previous study also showed that neighbourhood deprivation, gauged by an index of multiple deprivation (IMD), was a strong predictor for transitioning into a higher BMI group (Katsoulis et al., 2021). However, our study did not find similar evidence after examining other available SES measures (e.g., education, employment), indicating that further investigation is warranted to explore different SES measures associated with severe obesity development. Although an earlier study did not assess marital status and reported sex as a not significant predictor (Katsoulis et al., 2021), other research suggests sex and marital status may be important as the prevalence of severe obesity was generally higher among females (Booth et al., 2017; Zhao et al., 2023) and non‐married individuals (Zhao et al., 2023).

Our findings contribute to the existing psychosocial models of obesity (Tomiyama, 2014, 2019) and suggest the role of psychological well‐being may be more important in the development of obesity than its progression to severe obesity. The stronger link between poor psychological well‐being and weight gain in individuals without obesity suggests the importance of integrating psychological support into current existing obesity prevention policies and intervention programmes. However, it is important to note that these conclusions are limited to the specific psychological well‐being‐related measures examined in the present study and future research will benefit from exploring a broader range of measures to provide a more comprehensive perspective.

### Strengths and limitations

We used comparable national birth cohort studies that featured larger analytical sample sizes and multiple waves of data collection on BMI and measures related to psychological well‐being. The full‐analytical sample sizes in each cohort (*n* > 14,000) and pooled analyses (*n* > 33,000) had adequate analytical power to detect significance for relatively rare severe obesity development (3%, 1210–1540 cases in total across pooled analyses) in this study, which is a strength given that severe obesity cases in other studies tend to be limited in sample size (Chang et al., 2017; Robert et al., 2024). Limitations include a focus on three important but not extensive measures of psychological well‐being in the study, indicating future research will benefit from exploring the role of a wide range of other psychological well‐being‐related measures (e.g., positive affect, negative affect, loneliness, as in Putra et al. (2024) in the development of severe obesity. Both cohorts are characterized by a small percentage of participants recruited from ethnic minorities. Therefore, research in more ethnically diverse participants would now be informative. In most NCDS and BCS waves, BMI was derived from only available self‐reports of weight and height. However, self‐reported BMI is an accurate measure and is strongly correlated with objectively measured BMI in the general population (Hodge et al., 2020).

## CONCLUSION

We found limited evidence that psychological well‐being measures prospectively predict the development of severe obesity. Poorer psychological well‐being‐related measures were consistently associated with increased BMI in young and middle‐aged adults without obesity, but not in those with obesity.

## AUTHOR CONTRIBUTIONS

**I Gusti Ngurah Edi Putra:** Conceptualization; methodology; investigation; writing – original draft; formal analysis; project administration. **Sam Wilkinson:** Writing – original draft. **Michael Daly:** Conceptualization; methodology; supervision; writing – review and editing. **Eric Robinson:** Conceptualization; funding acquisition; writing – review and editing; methodology; supervision; investigation.

## FUNDING INFORMATION

Economic and Social Research Council (ESRC), the United Kingdom Research and Innovation (UKRI) (ES/V017594/1).

## CONFLICT OF INTEREST STATEMENT

The authors declared no conflict of interest.

## Supporting information


Table S1.–S8.

## Data Availability

The datasets used in this study are available online (NCDS: https://doi.org/10.5255/UKDA‐Series‐2000032, BCS: https://doi.org/10.5255/UKDA‐Series‐200001) with the permission of the UK Data Service.
