# Supplementary material for: Risk of severe obesity development: Examining the role of psychological well‐being related measures and sociodemographic factors in two longitudinal UK cohort studies
Source: Br J Health Psychol. 2025 Apr 21;30(2):e12798. doi: 10.1111/bjhp.12798 (PMC12010312; doi:10.1111/bjhp.12798)
Supplement: Supplementary file 1 — Table S1.–S8. [file BJHP-30-0-s001.pdf]

Supplementary materials

Table S1. Associations between individual psychological well-being related measures and severe obesity development and residualised BMI change scores using a pooled analytical sample size from both the 1958 NCDS and the 1970 BCS, controlling for sociodemographic covariates

Psychological well-being related measures	Severe obesity development vs. no development				Changes in BMI			
	**n**	**OR**	**95% CI**	**p-value**	**n**	**β**	**95% CI**	**p-value**
Depressive symptoms	46,201	0.99	0.94, 1.06	0.855	46,201	0.04	0.02, 0.06	0.001*
Life satisfaction	38,264	0.97	0.90, 1.03	0.319	38,264	-0.06	-0.08, -0.03	<0.001*
Self-efficacy	33,018	0.99	0.93, 1.06	0.853	33,018	-0.04	-0.07, -0.01	0.010*

**p-value remained statistically significant after correcting multiple comparisons (see Table S8)*

Only participants with no severe obesity (BMI 18.5 – <35 kg/m^2) at baseline were included.

n=number of observations; OR = odds ratio; β = regression coefficient; CI = confidence intervals

All psychological well-being related measures were in z-scores (mean = 0; SD = 1)

Separate regression models were developed for each psychological well-being related measure, adjusted for age, sex, ethnicity, marital status, childhood SES (or father's occupation), participant's occupation, education, housing tenure, baseline BMI (for the categorical outcome), and the study cohort.

Table S2. Interactions between individual psychological well-being related measures and the study cohort in predicting severe obesity development and residualised BMI change scores using a pooled analytical sample size from both the 1958 NCDS and the 1970 BCS, controlling for sociodemographic covariates

**Psychological well-being related measures * Cohort (BCS vs. NCDS)**	**Severe obesity development vs. no development**				**Changes in BMI**			
	**n**	**OR**	**95% CI**	**p-value**	**n**	**β**	**95% CI**	**p-value**
Depressive symptoms * BCS	46,201	0.98	0.87, 1.09	0.673	46,201	0.04	-0.01, 0.09	0.112
Life satisfaction * BCS	38,264	1.00	0.88, 1.15	0.971	38,264	-0.04	-0.09, 0.02	0.178
Self-efficacy * BCS	33,018	1.00	0.87, 1.14	0.980	33,018	-0.05	-0.11, 0.00	0.065

Only participants with no severe obesity (BMI 18.5 – <35 kg/m^2) at baseline were included.

n=number of observations; OR = odds ratio; β = regression coefficient; CI = confidence intervals

All psychological well-being related measures were in z-scores (mean = 0; SD = 1)

Separate regression models were developed for each psychological well-being related measure, adjusted for age, sex, ethnicity, marital status, childhood SES (or father's occupation), participant's occupation, education, housing tenure, baseline BMI (for the categorical outcome), and the study cohort.

Table S3. Associations between individual psychological well-being related measures and severe obesity development and residualised BMI change scores using a pooled analytical sample size from both the 1958 NCDS and the 1970 BCS for a longer follow-up, controlling for sociodemographic covariates

**Psychological well-being related measures**	**NCDS: Baseline: age 33, follow-up: age 50 BCS: Baseline: age 30, follow-up: age 46**							
	**Severe obesity development vs. no development**				**Changes in BMI**			
	**n**	**OR**	**95% CI**	**p-value**	**n**	**β**	**95% CI**	**p-value**
Depressive symptoms	11,535	1.07	0.99, 1.16	0.085	11,535	0.15	0.08, 0.21	<0.001*
Life satisfaction	11,535	0.90	0.83, 0.98	0.012*	11,535	-0.12	-0.18, -0.06	<0.001*
Self-efficacy	11,535	0.97	0.90, 1.05	0.479	11,535	-0.09	-0.15, -0.03	0.006*

**p-value remained statistically significant after correcting multiple comparisons (see Table S8)*

Only participants with no severe obesity (BMI 18.5 – <35 kg/m^2) at baseline were included.

n=number of observations; OR = odds ratio; β = regression coefficient; CI = confidence intervals

All psychological well-being related measures were in z-scores (mean = 0; SD = 1)

Separate regression models were developed for each psychological well-being related measure, adjusted for age, sex, marital status, childhood SES (or father's occupation), participant's occupation, education, housing tenure, baseline BMI (for the categorical outcome), and the study cohort. Ethnicity was not controlled for due to a small percentage of participants from ethnic minorities.

Table S4. Interactions between individual psychological well-being related measures and the study cohort in predicting severe obesity development and residualised BMI change scores using a pooled analytical sample size from both the 1958 NCDS and the 1970 BCS for a longer follow-up, controlling for sociodemographic covariates

**Psychological well-being related measures * Cohort (BCS vs. NCDS)**	**NCDS: Baseline: age 33, follow-up: age 50 BCS: Baseline: age 30, follow-up: age 46**							
	**Severe obesity development vs. no development**				**Changes in BMI**			
	**n**	**OR**	**95% CI**	**p-value**	**n**	**β**	**95% CI**	**p-value**
Depressive symptoms * BCS	11,535	1.03	0.88, 1.20	0.742	11,535	0.16	0.04, 0.28	0.009*
Life satisfaction * BCS	11,535	1.08	0.92, 1.27	0.349	11,535	-0.14	-0.26, -0.02	0.027
Self-efficacy * BCS	11,535	1.11	0.95, 1.30	0.198	11,535	-0.04	-0.17, 0.08	0.481

**p-value remained statistically significant after correcting multiple comparisons (see Table S8)*

Only participants with no severe obesity (BMI 18.5 – <35 kg/m^2) at baseline were included.

n=number of observations; OR = odds ratio; β = regression coefficient; CI = confidence intervals

All psychological well-being related measures were in z-scores (mean = 0; SD = 1)

Separate regression models were developed for each psychological well-being related measure, adjusted for age, sex, marital status, childhood SES (or father's occupation), participant's occupation, education, housing tenure, baseline BMI (for the categorical outcome), and the study cohort. Ethnicity was not controlled for due to a small percentage of participants from ethnic minorities.

Table S5. Associations between all the psychological well-being related measures in concert and residualised BMI change scores using a pooled analytical sample size from both the 1958 NCDS and the 1970 BCS for a longer follow-up, controlling for sociodemographic covariates

**Psychological well-being related measures**	**NCDS: Baseline: age 33, follow-up: age 50<br>BCS: Baseline: age 30, follow-up: age 46**			
	**Changes in BMI**			
	**n**	**β**	**95% CI**	**p-value**
Depressive symptoms	11,535	0.12	0.05, 0.19	0.001*
Life satisfaction	11,535	-0.07	-0.15, -0.00	0.050
Self-efficacy	11,535	-0.01	-0.08, 0.06	0.789

**p-value remained statistically significant after correcting multiple comparisons (see Table S8)*
Only participants with no severe obesity (BMI 18.5 – < 35 kg/m^2) at baseline were included.
n=number of observations; OR = odds ratio; β = regression coefficient; CI = confidence intervals
All psychological well-being related measures were in z-scores (mean = 0; SD = 1)
All the psychological well-being related measures were included in the same regression model, adjusted for age, sex, marital status, childhood SES (or father's occupation), participant's occupation, education, housing tenure, and the study cohort. Ethnicity was not controlled for due to a small percentage of participants from ethnic minorities.

Table S6. Sociodemographic characteristics associated with severe obesity development using a pooled analytical sample size from both the 1958 NCDS and the 1970 BCS

**Sociodemographic characteristics**	**Severe obesity development vs. no development (n = 46,201)**			
	**OR**	**95% CI**	**p-value**	**Overall p-value**
Age *(ref: 23)*				
26	0.17	0.12, 0.25	<0.001	<0.001*
30	0.19	0.14, 0.27	<0.001	
33	0.15	0.11, 0.19	<0.001	
34	0.20	0.15, 0.28	<0.001	
42	0.33	0.26, 0.43	<0.001	
Sex (*ref: Male)*				
Female	1.77	1.55, 2.03	<0.001	<0.001*
Ethnicity *(ref: Non-White)*				
White	1.51	0.54, 4.21	0.433	0.433
Marital status *(ref: Married)*				
Single	1.33	1.15, 1.55	<0.001	<0.001*
Others	1.31	1.06, 1.63	0.013	
Father's occupation *(ref: Professional)*				
Intermediate	0.96	0.66, 1.38	0.815	0.079
Skilled	1.08	0.75, 1.54	0.682	
Partly skilled	1.28	0.88, 1.87	0.193	
Unskilled	1.24	0.80, 1.91	0.330	
Others	1.42	0.89, 2.29	0.143	
CM's occupation (*ref: Professional)*				
Intermediate	1.08	0.75, 1.56	0.687	0.663
Skilled	1.23	0.84, 1.79	0.292	
Partly skilled	1.20	0.80, 1.80	0.392	
Unskilled	1.10	0.65, 1.85	0.729	
Others	1.22	0.82, 1.84	0.323	
CM's education *(ref: NVQ level 5)*				
No qualification	0.94	0.67, 1.33	0.738	0.847
NVQ level 1	0.97	0.69, 1.38	0.875	
NVQ level 2	0.88	0.65, 1.21	0.452	
NVQ level 3	0.97	0.70, 1.35	0.859	
NVQ level 4	0.88	0.65, 1.20	0.422	
Housing tenure *(ref: Others)*				
Owner-occupier	0.85	0.73, 1.00	0.047	0.047

**p-value remained statistically significant after correcting multiple comparisons (see Table S8)*

Only participants with no severe obesity (BMI 18.5 – < 35 kg/m^2) at baseline were included.

n=number of observations; OR = odds ratio; CI = confidence intervals

Overall p-values for independent categorical variables with more than two values or categories were obtained using a post-estimation test, "mi test" command. These p-values were then corrected using the Benjamini-Hochberg (BH) adjustment method (see Table S8)

All the sociodemographic characteristics (age, sex, marital status, childhood SES (or father's occupation), participant's occupation, education, housing tenure) were included in the same regression model, controlling for baseline BMI and the study cohort.

Table S7. Associations between individual psychological well-being related measures and residualised BMI change scores using a pooled analytical sample size from both the 1958 NCDS and the 1970 BCS stratified by baseline BMI categories, controlling for sociodemographic covariates

**Psychological well-being-related measures**	**Changes in BMI**							
	**Non-obesity (BMI 18.5 – <30)**				**Non-severe obesity (BMI 30 – <35)**			
	**n**	**β**	**95% CI**	**p-value**	**n**	**β**	**95% CI**	**p-value**
Depressive symptoms	42,234	0.05	0.02, 0.08	<0.001*	3,967	-0.08	-0.20, 0.04	0.214
Life satisfaction	34,488	-0.07	-0.10, -0.05	<0.001*	3,776	0.08	-0.04, 0.19	0.196
Self-efficacy	29,521	-0.05	-0.08, -0.02	0.001*	3,497	0.07	-0.05, 0.19	0.252

**p-value remained statistically significant after correcting multiple comparisons (see Table S8)*
Only participants with no severe obesity (BMI 18.5 – <35 kg/m²) at baseline were included.
n=number of observations; OR = odds ratio; β = regression coefficient; CI = confidence intervals
All psychological well-being related measures were in z-scores (mean = 0; SD = 1)
Separate regression models were developed for each psychological well-being related measure, adjusted for age, sex, ethnicity, marital status, childhood SES (or father's occupation), participant's occupation, education, housing tenure, and the study cohort.

Table S8. Correction for multiple testing using the Benjamini-Hochberg (BH) adjustment method

Tables	Cohort studies - Independent - dependent variables	p-value	Rank	(i/n)q
3	BCS - Life satisfaction - Changes in BMI	<0.001	1	0.001
S1	NCDS&BCS - Life satisfaction - Changes in BMI	<0.001	2	0.002
5	BCS - Depressive symptoms - Changes in BMI	<0.001	3	0.003
S3	NCDS&BCS - Depressive symptoms - Changes in BMI	<0.001	4	0.003
S3	NCDS&BCS - Life satisfaction - Changes in BMI	<0.001	5	0.004
S6	NCDS&BCS - Age	<0.001	6	0.005
S6	NCDS&BCS - Sex	<0.001	7	0.006
S6	NCDS&BCS - Marital status	<0.001	8	0.007
S7	NCDS&BCS - Depressive symptoms - Changes in BMI (non-OB)	<0.001	9	0.008
S7	NCDS&BCS - Life satisfaction - Changes in BMI (non-OB)	<0.001	10	0.008
3	BCS - Depressive symptoms - Changes in BMI	0.001	11	0.009
S1	NCDS&BCS - Depressive symptoms - Changes in BMI	0.001	12	0.010
5	BCS - Life satisfaction - Changes in BMI	0.001	13	0.011
S5	NCDS&BCS - Depressive symptoms - Changes in BMI	0.001	14	0.012
S7	NCDS&BCS - Self-efficacy - Changes in BMI (non-OB)	0.001	15	0.013
5	NCDS - Depressive symptoms - Changes in BMI	0.002	16	0.014
3	BCS - Self-efficacy - Changes in BMI	0.003	17	0.014
S3	NCDS&BCS - Self-efficacy - Changes in BMI	0.006	18	0.015
S4	NCDS&BCS - Depressive symptoms - Changes in BMI	0.009	19	0.016
S1	NCDS&BCS - Self-efficacy - Changes in BMI	0.010	20	0.017
4	NCDS - Life satisfaction - Severe obesity development	0.012	21	0.018
S3	NCDS&BCS - Life satisfaction - Severe obesity development	**0.012**	**22**	**0.019**
5	NCDS - Self-efficacy - Changes in BMI	0.019	23	0.019
S4	NCDS&BCS - Life satisfaction - Changes in BMI	0.027	24	0.020
S6	NCDS&BCS - Housing tenure	0.047	25	0.021
3	NCDS - Life satisfaction - Changes in BMI	0.050	26	0.022
S5	NCDS&BCS - Life satisfaction - Changes in BMI	0.050	27	0.023
4	NCDS - Depressive symptoms - Severe obesity development	0.060	28	0.024
S2	NCDS&BCS - Self-efficacy - Changes in BMI	0.065	29	0.025
4	NCDS - Self-efficacy - Severe obesity development	0.070	30	0.025
S6	NCDS&BCS - Father's occupation	0.079	31	0.026
5	BCS - Self-efficacy - Changes in BMI	0.080	32	0.027
S3	NCDS&BCS - Depressive symptoms - Severe obesity development	0.085	33	0.028
S2	NCDS&BCS - Depressive symptoms - Changes in BMI	0.112	34	0.029
3	NCDS - Depressive symptoms - Changes in BMI	0.117	35	0.030
5	NCDS - Life satisfaction - Changes in BMI	0.120	36	0.031
S2	NCDS&BCS - Life satisfaction - Changes in BMI	0.178	37	0.031
S7	NCDS&BCS - Life satisfaction - Changes in BMI	0.196	38	0.032
S4	NCDS&BCS - Self-efficacy - Severe obesity development	0.198	39	0.033
S7	NCDS&BCS - Depressive symptoms - Changes in BMI	0.214	40	0.034
S7	NCDS&BCS - Self-efficacy - Changes in BMI	0.252	41	0.035
4	BCS - Life satisfaction - Severe obesity development	0.283	42	0.036

4	BCS - Depressive symptoms - Severe obesity development	0.294	43	0.036
S1	NCDS&BCS - Life satisfaction - Severe obesity development	0.319	44	0.037
S4	NCDS&BCS - Life satisfaction - Severe obesity development	0.349	45	0.038
S6	NCDS&BCS - Ethnicity	0.433	46	0.039
S3	NCDS&BCS - Self-efficacy - Severe obesity development	0.479	47	0.040
S4	NCDS&BCS - Self-efficacy - Changes in BMI	0.481	48	0.041
3	NCDS - Self-efficacy - Changes in BMI	0.571	49	0.042
4	BCS - Self-efficacy - Severe obesity development	0.590	50	0.042
S6	NCDS&BCS - Occupation	0.663	51	0.043
S2	NCDS&BCS - Depressive symptoms - Severe obesity development	0.673	52	0.044
S4	NCDS&BCS - Depressive symptoms - Severe obesity development	0.742	53	0.045
S5	NCDS&BCS - Self-efficacy - Changes in BMI	0.789	54	0.046
S6	NCDS&BCS - Education	0.847	55	0.047
S1	NCDS&BCS - Self-efficacy - Severe obesity development	0.853	56	0.047
S1	NCDS&BCS - Depressive symptoms - Severe obesity development	0.855	57	0.048
S2	NCDS&BCS - Life satisfaction - Severe obesity development	0.971	58	0.049
S2	NCDS&BCS - Self-efficacy - Severe obesity development	0.980	59	0.050

i = the individual p-value's rank; n = total number of tests (59); q = the false discovery rate (5%)

The largest p-value that is lower than the Benjamini-Hochberg critical value is 0.012 (Rank 22). This p-value and all smaller p-values will be considered statistically significant.
